# Non-Specific Antibodies Induce Lysosomal Activation in Atlantic Salmon Macrophages Infected by *Piscirickettsia salmonis*

**DOI:** 10.3389/fimmu.2020.544718

**Published:** 2020-11-12

**Authors:** Diego Pérez-Stuardo, Allison Espinoza, Sebastián Tapia, Jonathan Morales-Reyes, Claudio Barrientos, Eva Vallejos-Vidal, Ana M. Sandino, Eugenio Spencer, Daniela Toro-Ascuy, J. Andrés Rivas-Pardo, Felipe E. Reyes-López, Sebastián Reyes-Cerpa

**Affiliations:** ^1^Centro de Genómica y Bioinformática, Facultad de Ciencias, Universidad Mayor, Santiago, Chile; ^2^Consorcio Tecnológico de Sanidad Acuícola, Ictio Biotechnologies S.A., Santiago, Chile; ^3^Department of Cell Biology, Physiology and Immunology, Universitat Autonoma de Barcelona, Bellaterra, Spain; ^4^Centro de Biotecnología Acuícola, Facultad de Química y Biología, Universidad de Santiago de Chile, Santiago, Chile; ^5^Laboratorio de Virología, Instituto de Ciencias Biomedicas, Facultad de Ciencias de la Salud, Universidad Autónoma de Chile, Santiago, Chile; ^6^Escuela de Biotecnología, Facultad de Ciencias, Universidad Mayor, Santiago, Chile

**Keywords:** macrophages, Atlantic salmon, IgM, *P. salmonis*, lysosome activity

## Abstract

*Piscirickettsia salmonis*, an aggressive intracellular pathogen, is the etiological agent of salmonid rickettsial septicemia (SRS). This is a chronic multisystemic disease that generates high mortalities and large losses in Chilean salmon farming, threatening the sustainability of the salmon industry. Previous reports suggest that *P. salmonis* is able to survive and replicate in salmonid macrophages, inducing an anti-inflammatory environment and a limited lysosomal response that may be associated with host immune evasion mechanisms favoring bacterial survival. Current control and prophylaxis strategies against *P. salmonis* (based on the use of antibiotics and vaccines) have not had the expected success against infection. This makes it urgent to unravel the host-pathogen interaction to develop more effective therapeutic strategies. In this study, we evaluated the effect of treatment with IgM-beads on lysosomal activity in Atlantic salmon macrophage-enriched cell cultures infected with *P. salmonis* by analyzing the lysosomal pH and proteolytic ability through confocal microscopy. The impact of IgM-beads on cytotoxicity induced by *P. salmonis* in infected cells was evaluated by quantification of cell lysis through release of Lactate Dehydrogenase (LDH) activity. Bacterial load was determined by quantification of *16S* rDNA copy number by qPCR, and counting of colony-forming units (CFU) present in the extracellular and intracellular environment. Our results suggest that stimulation with antibodies promotes lysosomal activity by lowering lysosomal pH and increasing the proteolytic activity within this organelle. Additionally, incubation with IgM-beads elicits a decrease in bacterial-induced cytotoxicity in infected Atlantic salmon macrophages and reduces the bacterial load. Overall, our results suggest that stimulation of cells infected by *P. salmonis* with IgM-beads reverses the modulation of the lysosomal activity induced by bacterial infection, promoting macrophage survival and bacterial elimination. This work represents a new important evidence to understand the bacterial evasion mechanisms established by *P. salmonis* and contribute to the development of new effective therapeutic strategies against SRS.

## Introduction

*Piscirickettsia salmonis* is the etiological agent of piscirickettsiosis or salmonid rickettsial septicemia (SRS), which mostly affects farmed salmonid species (1, 2). *Piscirickettsia salmonis* is a Gram-negative, non-motile, unencapsulated, pleomorphic, and usually coccoid bacterium, between 0.2 and 1.5 μm in diameter (1, 3, 4). It is an intracellular pathogen, classified phylogenetically as a *Gammaproteobacteria* in the family *Piscirickettsiaceae*, and closely related order to *Legionella*, *Francisella*, and *Coxiella* (1).

In Chile, the National Fisheries Service (SERNAPESCA, Servicio Nacional de Pesca) has identified SRS as the most serious health problem facing the Chilean salmon industry (5) owing to its highly aggressive nature, recurrent outbreaks, and widespread transmission among other cultivated salmonid species (6–9). In 2018, mortalities associated with *P. salmonis* represented 54.7% and 83.3% of the total mortalities attributed to infectious causes in Atlantic salmon (*Salmo salar* L.) and rainbow trout (*Oncorhynchus mykiss*), respectively (9). The control and prophylactic strategies against *P. salmonis* have relied on antibiotics and vaccines to date; however, both are inadequate. Antibiotics have been used indiscriminately to control outbreaks of infection. In 2018, the Chilean aquaculture industry alone utilized over 322 tons of antibiotics, mainly florfenicol and oxytetracycline (10). Moreover, infected salmonids respond poorly to these treatments, likely because of the intracellular characteristics of the infective cycle of *P. salmonis* and the insufficient concentrations of antibiotics that reach the intracellular niche to eliminate the bacterium (11). This situation is further complicated by the lack of effective vaccines against *P. salmonis* (12), because prophylactic vaccines do not provide acceptable levels of protection (11).

Despite the severe impact of *P. salmonis* on the Chilean aquaculture industry, key aspects of this bacterium, such as its life cycle and pathogenic mechanisms, are poorly characterized (13). It is reported that *P. salmonis* survives and replicates inside macrophage vacuoles (14) that do not mature to fuse them with lysosomes to degrade the pathogen (13, 15). Recently, we observed that macrophages infected by *P. salmonis* have a lower number of lysosomes in comparison with those incubated with inactivated *P. salmonis*; as a consequence, these infected macrophages exhibited fewer proteolytic foci and also had a pH close to neutral (16). Moreover, it has been suggested that *P. salmonis* induces an anti-inflammatory milieu when it infects macrophages, by manipulating the host cytokine profile to promote an environment that is favorable for its survival and replication in salmonid macrophage-like cells (17).

The low effectiveness of antibiotic treatment is not only observed against *P. salmonis*, but also against other intracellular pathogens, such as *Legionella pneumophila*, for which erythromycin (antibiotic most commonly used for 25 years to treat infections with this bacterium) is no longer effective in *in vivo* models (18). In this scenario, the passive immunization strategy against infection with *L. pneumophila* proposed by Joller et al. (19) offers new prospects in the development of antimicrobials against intracellular pathogens. Joller et al. used non-specific antibodies that form IgG-beads that stimulate the intracellular immune response in macrophages infected with *L. pneumophila*, promoting their degradation within lysosomes of infected cells. The underlying mechanism is suggested to involve induction of the signaling cascade that counteracts the modulation of the endocytic vacuolar traffic (19). The activation of the response of cells infected by *L. pneumophila* due to the IgG-beads stimulation is dependent on the interaction of the Fc region of non-specific antibodies with the Fc receptor (FcR) present in macrophages (20).

In salmonids, antibody-mediated opsonization of particles enhances phagocytosis and respiratory burst by phagocytes, providing functional evidence for the presence of antibody receptors on leukocytes (21–24). In Atlantic salmon, this mechanism is potentially mediated by receptors that may be similar to Fc receptors, which are widely distributed on leukocytes in mammals (25, 26). The utilization of antibodies bound to antigens as a passive immunization strategy against infectious diseases has been poorly explored. In the context of strategies against bacteria such as *Flavobacterium psychrophilum* and *Vibrio anguillarum* in rainbow trout, the delivery of specific antibodies attenuates the mortality rates and aggressiveness associated with infectious outbreaks, suggesting a protective role and a putative immune stimulatory effect (27, 28).

Previously, our group reported that *P. salmonis* survives at least 120 h within Atlantic salmon macrophages-enriched cell cultures. During this time, the intracellular bacterial load rises, as evidenced by an increase in the number of copies of the bacterial *16S* rDNA ribosomal gene. Additionally, we suggest that the survival of the *P. salmonis* is favored by perturbations in lysosomal activity, as evidenced by a limited lysosomal proteolytic activity observed in infected cells (16). The aim of this study was to evaluate whether the use of non-specific antibodies forming IgM-beads activate the lysosomal response of macrophage-enriched cell cultures infected by *P. salmonis*. The effectiveness of the treatment was analyzed in terms of the effect that non-specific antibodies forming IgM-beads would have on the modulation of both lysosomal acidification and proteolytic activity induced by the bacterium when it infects macrophages-like cells of Atlantic salmon. Additionally, we evaluated the effect on bacterial load and survival of infected macrophages.

## Materials and Methods

### Experimental Fish

Atlantic salmon with an average weight of 55 ± 15 g were obtained from a local farm and maintained in tanks with freshwater at a biomass of 10–12 kg/m^3^, with controlled temperature (14–16°C) and continuous aeration. Water quality parameters, such as oxygen, pH, and levels of nitrogen compounds (i.e., nitrate, nitrite, and ammonia) were monitored daily and maintained at constant values. The fish were fed with commercial pellet twice daily (Golden Optima, Biomar, Puerto Montt, Chile), and acclimatized for 3 weeks prior to the experiments. The maintenance of fish was performed in accordance with the ethical standards of the Institutional Ethics Committee of Universidad de Santiago de Chile (approved in internal report n°364) and the relevant legislation in force.

### Isolation of Macrophages and Cell Cultures

Macrophage-enriched cell cultures were obtained from Atlantic salmon head kidneys, as described by Braun-Nesje et al. (29) and by our group in Pérez-Stuardo et al. (16) with slight modifications. Briefly, Atlantic salmon head kidney were aseptically extracted and disaggregated using a cell strainer (pore size: 70 µm) (BD Falcon, Seaton Delaval, England) suspended in Leibovitz-15 medium (L-15; Corning, New York, USA) with supplement 1 (**Supplementary Table 1**). Mechanical disaggregation was performed until a homogeneous cell suspension was obtained. The leukocyte fraction was isolated through a discontinuous gradient in densities of 34 and 51% of Percoll (GE Healthcare) diluted in miliQ water, supplemented with 1× Hank’s Balanced Salt Solution (HBSS, Gibco). The cell suspension was placed on the Percoll gradient, and centrifuged at 400 g for 40 min at 16°C. The leukocyte interface was collected and resuspended in L-15 medium with supplement 1 (**Supplementary Table 1**). To eliminate the traces of Percoll, the cell suspension was centrifuged twice for 7 min at 250 g at 16°C. The cell suspension was seeded at 40,000 cells/cm^2^ in L-15 medium with supplement 2 (**Supplementary Table 1**) at 16°C.

To enrich the primary cell culture with monocytic/macrophage adherent cell population, the primary culture was washed the following day with three washes of 1× phosphate-buffered saline (PBS) at pH 7.4. Non-adherent cells were discarded and the remaining cells were cultivated with fresh L-15 medium with supplement 2 (**Supplementary Table 1**). At 3, 5, and 7 days of cultivation, the cells were washed three times with 1× PBS. Non-adherent cells were discarded, and fresh L-15 medium with supplement 3 (**Supplementary Table 1**) was added to the cultures.

SHK-1 cells (*Salmo salar*; ATCC, American Type Culture Collection, Manassas, VA, USA), which are described as macrophage-like cells (30), were grown at 16°C in L-15 medium with supplement 4 (**Supplementary Table 1**).

### *Piscirickettsia salmonis* and Infection

Culture and propagation of *P. salmonis* (strain 9734, ETECMA, Puerto Montt, Chile) was performed in salmonid cell line CHSE-214 (ATCC N°CRL-1682), as previously described by Fryer et al., 1992 (30). The CHSE-214 cell line was maintained in minimal essential medium (MEM; Corning) with supplement 5 (**Supplementary Table 1**) at 16°C. The infected cells were observed using conventional inverted light microscopy (Motic AE31E, Leica Microsystems, Wetzlar, Germany) after 4 to 6 days post-infection (dpi) to observe any cytopathic effect (CPE) (31).

*Piscirickettsia salmonis* was extracted from the supernatant of infected CHSE-214 cells displaying CPE. Bacterial quantification was performed in a Petroff-Hausser chamber (Hausser Scientific, PA, USA) according to the instructions provided by the manufacturer. Cellular debris was eliminated through centrifugation for 5 min at 500 g. Subsequently, *P. salmonis* was centrifuged at 7,500 *g* for 15 min at 16°C. Macrophage-enriched cell cultures from Atlantic salmon head kidneys were seeded at 6,000 cells/cm^2^ in six-well flat bottom plates and were incubated for 2 h with *P. salmonis* at a multiplicity of infection (MOI) of 10 bacteria/cell in L-15 medium with supplement 3 (**Supplementary Table 1**). Infection was synchronized by centrifugation at 150 g for 3 min at 16°C, and the infected cells were incubated at 16°C. Then, cells were washed twice with 1× PBS and incubated with L-15 medium with supplement 3 (**Supplementary Table 1**). As a control, *P. salmonis* was inactivated by incubation of the pellet in 4% paraformaldehyde (PFA; Sigma Aldrich, MO, USA), diluted in 1× PBS and incubated overnight at 4°C. The bacterial suspension was centrifuged for 15 min at 7,500 *g* at 16°C; then, the supernatant was discarded and the pellet was washed three times with 1× PBS. Finally, the bacterial suspension was centrifuged for 15 min at 7,500 g at 16°C; the supernatant was discarded and the pellet was resuspended in L-15 medium with supplement 3 (**Supplementary Table 1**).

### Evaluation of Lysosomal Acidification

Evaluation of lysosomal acidification in macrophage-enriched cell cultures was performed by fluorescence analysis using the LysoSensor™ Yellow/Blue probe (LSYB; Thermo Scientific). This ratiometric probe can be used to measure the pH of acidic organelles. The LysoSensor™ dye produces blue and yellow fluorescence in neutral and acidic environments, respectively. Therefore, a fluorescence shift from blue (maximum emission at 430 nm) to yellow (maximum emission at 535 nm) indicates a decrease in lysosomal pH (16, 32).

The IgM-beads were prepared with latex beads coated with immunoglobulin M (IgM). IgM was purified from the serum of Atlantic salmon (weighing 80 to 100 g) by an affinity protein A sepharose column. The presence of IgM was confirmed by western blotting and ELISA based on a monoclonal antibody (I-14 hybridoma) (33). Additionally, the homogeneity of the IgM preparation was evaluated by SDS-PAGE. In order to assess the short-term effect of the IgM-beads on lysosomal acidification, the macrophage-enriched cell cultures were seeded at 6,000 cells/cm^2^ in 12-well flat bottom plates with glass coverslips and infected with *P. salmonis* at MOI 10 for 2 h, as previously described. Then, infected macrophage-enriched cell culture was incubated for 1 h with 30 beads/cell. After incubation, the cells were washed twice with 1× PBS and cultivated in fresh L-15 medium with supplement 3 (**Supplementary Table 1**). The analysis of lysosomal acidification was performed at 1 and 3 h post-treatment (hpt), according to the described by Pérez-Stuardo et al. (16). Additionally, we evaluated the effect of Bovine Serum Albumin (BSA)-beads in infected cells as protein-beads control. To evaluate the lysosomal acidification in macrophage-enriched cell cultures infected by *P. salmonis*, we incubated the cells with bacteria for 2 h as was previously described and maintained in L-15 medium with supplement 3 for 3 and 5 h post-infection (hpi) (equivalent to 1 and 3 hpt). Similarly, as a control for lysosomal activation, macrophage-enriched cell cultures were incubated with inactivated *P. salmonis* and evaluated at the same times as the experimental group.

At each time point, the infected cells were incubated for 5 min with 10 µM LSYB. Subsequently, the cells were washed three times with 1× PBS and fixed for 10 min with 4% (w/v) PFA (Sigma Aldrich), followed by three additional washes with 1× PBS. Finally, the cells were stained by incubating for 1 min with 1 μg/mL propidium iodide (PI); then, the cells were washed twice with 1× PBS and once with MilliQ water to remove the residual salts. The samples were mounted on slides with Fluoromount™ (Sigma Aldrich), and images were obtained using a Leica SP8 confocal microscope (Leica, Wetzlar, Germany). Results were obtained by the division of the emission spectrum of the LSYB probe, specifically to obtain an acidic indicator channel (acquired between 500 and 580 nm) and a neutral-basic indicator channel (acquired between 450 and 495 nm). According to instructions provided by the manufacturer. The fluorescence intensity obtained in the acidic indicator channel had to be ≥2-fold that obtained in the neutral-basic indicator channel for a lysosome to be considered as acidic. The analysis of lysosomal acidity was performed using the software LAS X (version 3.3.0). Briefly, the average fluorescence intensity for each indicator channel was obtained for each lysosome present in the micrographs obtained from the samples. Subsequently, the ratio between the fluorescence intensity values obtained from the two indicator channels was used as the acidity index. An acidic index of x≥2 denoted an acidic pH, while an acidity index between 0<x<2 represented a neutral-basic pH. The number of lysosomes per sample was normalized to the number of cells per sample. For each condition, four random micrographs were obtained with a z-stack containing 30 cells mean. The micrographs were processed using the software Fiji (ImageJ 1.52g) (34). The results are represented as the number of lysosomes/cell and percentage of acidic lysosomes per condition.

### Evaluation of Lysosomal Activity

In order to assess the short-term effect on the lysosomal activity in *P. salmonis*-infected macrophage-enriched cell cultures, fluorescence analysis using the DQ™ Green BSA probe was performed in a similar way as previously described by our group in Pérez-Stuardo et al. (16). The DQ™ Green BSA probe is composed of albumin derivatized with a self-quenching fluorochrome. The degradation of DQ™ Green BSA in acidic lysosomes results in smaller protein fragments than those of isolated fluorophores. Once the quencher is released, brightly fluorescent products are observed. The cleavage of DQ™ Green BSA results in the release of fragments with maximum excitation and emission at 505 and 515 nm, respectively (35–37).

The macrophage-enriched cell cultures were established at 6,000 cells/cm^2^ in 12-well flat bottom plates with glass coverslips and infected with *P. salmonis*, as described in the previous section. Subsequently, analysis of lysosomal activity was performed at 1 and 3 hpt from IgM-beads incubation. The macrophage-enriched cells were treated with IgM-beads (or BSA-beads as control) in the same way as described in the previous section. Lysosomal activity was also evaluated in infected macrophage-enriched cell cultures incubated with BSA-beads. To evaluate the lysosomal activity in macrophage-enriched cell cultures infected by *P. salmonis*, we incubated the cells with bacteria for 2 h as was previously described and maintained in L-15 medium containing supplement 3 for 3 and 5 hpi (equivalent to 1 and 3 hpt). As positive control for lysosomal activation, macrophage-enriched cell cultures were incubated with inactivated *P. salmonis*, and lysosomal activity was evaluated at the same times as in the experimental group.

Two hours prior to each time point for IgM-beads incubation, DQ™ Green BSA 10 µg/mL in L-15 medium containing supplement 3 (**Supplementary Table 1**) was added to the evaluated cells. Then, the cells were washed three times with 1× PBS and fixed using 4% (w/v) PFA, followed by three additional washes with 1× PBS. The cells were stained with 1 μg/mL PI as previously described. The samples were mounted on slides using the mounting solution Fluoromount™ (Thermo Scientific). Micrographs were obtained using a Leica SP 8 confocal microscope, and processed and analyzed using Fiji software (ImageJ 1.52g) (34). The analysis was performed by counting each positive event per cell due to the fluorescence of DQ™ Green BSA, from four micrographs, with a merge of the images from z-stack, containing an average of 30 cells per micrograph for each experimental condition. The data were normalized to the number of cells on the micrograph, and the results are represented as the number of proteolytic events/cell.

### Evaluation of the Effect of Non-specific Antibodies Forming IgM-Beads on Cytotoxicity Induced by *P. salmonis*

To evaluate the effect of IgM-beads on cytotoxicity induced by *P. salmonis*, we quantified lactate dehydrogenase (LDH) release into the extracellular medium. The macrophage-enriched cell cultures were seeded at 10,000 cells/well in 96-well flat bottom plates. Cells were incubated with *P. salmonis* at MOI 10 for 2 h, as previously described. Then, cells were incubated for 1 h with 30 IgM-beads/cell. After incubation, cells were washed twice with 1× PBS and cultivated in fresh L-15 medium with supplement 3 (**Supplementary Table 1**). Cytotoxicity was evaluated in macrophage-enriched cell culture at 3, 5, and 7 days post-treatment (dpt) using the Pierce LDH Cytotoxicity Assay Kit (Thermo Scientific), according to the instructions of the manufacturer. As the control, we evaluated the cytotoxicity induced by *P. salmonis* and the effect of BSA-beads and non-coupled-beads on infected cells.

### Gentamicin Protection Assay and Quantification of Bacterial Load

In order to recover intracellular bacteria from the infected macrophage-like cells, a gentamicin protection assay was performed. Briefly, macrophage-like cells (SHK-1 cell line) were seeded at a density of 150,000 cells/well in 6-well flat bottom plates. Cells were infected with *P. salmonis* at MOI 10 for 24 h. Infection was synchronized by centrifugation, and the infected cells were incubated at 16°C. Then, cells were washed twice with 1× PBS and incubated with L-15 medium with supplement 3 (**Supplementary Table 1**). SHK-1-infected cells were incubated with 30 IgM-beads/cell, and the infection was maintained until 72 and 120 hpi. Recovery of the intracellular bacterium was performed following the protocol described by Pérez-Stuardo et al. (16). As control, bacterial load was also evaluated in infected macrophage-enriched cell cultures incubated with BSA-beads.

To quantify the extracellular bacterial load, the supernatant from SHK-1 cells infected with *P. salmonis* was recovered and centrifuged for 15 min at 7,500 *g* at 16°C. The supernatant was discarded, and the pellet was resuspended in 1× PBS. Accordingly, the sample was subdivided to (i) isolate DNA in order to quantify the bacterial load by qPCR, and (ii) determine the bacterial viability by plating on serial dilutions (from 10^−1^ to 10^−5^) in CHAB agar which includes: Cystine heart agar [25 g/L Bacto heart Infusion broth (BD Difco), 10g/L Glucose (Merck), 1 g/L L-cysteine (Merck), 15 g/L agar (Sigma Aldrich), 2g/L hemoglobin from bovine blood (Sigma Aldrich)] supplemented with 5% bovine blood (Health Public Institute Chile) (38). To quantify the intracellular bacteria, macrophage-enriched cell cultures infected with *P. salmonis* were incubated with 100 μg/mL gentamicin for 60 min to eliminate bacteria from the extracellular environment. Subsequently, the cells were washed three times with cold 1× PBS, and incubated for 15 min with 1% (w/v) saponin (Sigma Aldrich) in 1× PBS at 16°C. Finally, the permeabilized cells were suspended in 1× PBS and centrifuged at 7,500 g for 10 min at 4°C. The supernatant was then discarded, and the pellet was resuspended in 1× PBS. The sample was subdivided to (i) isolate DNA in order to quantify the bacterial load by qPCR, and (ii) determine bacterial viability by plating on serial dilutions (from 10^−1^ to 10^−5^) in CHAB agar, as described above.

### Detection of *P. salmonis* Using Quantitative Polymerase Chain Reaction (qPCR)

The gene encoding *16S* rRNA (primers, Fw: 5’-AGG-GAG-ACT-GCC-GGT-GAT-A-3’; Rv: 5’-ACT-ACG-AGG-CGC-TTT-CTC-A-3’) was amplified as described by Karatas et al. (2008) in order to detect the presence of *P. salmonis* in the infected cell cultures (39). Genomic DNA was obtained using the Wizard™ Genomic DNA Purification kit (Promega, WI, USA) according to the instructions provided by the manufacturer. PCR amplification was performed using the PowerUp™ SYBR^®^ Green Master Mix (Thermo Scientific) according to the manufacturer’s instructions. The primers were added to a final concentration of 0.4 µM, and 12 ng of template was used. The qPCR was performed on a QuantStudio 3 Real-Time PCR system (Thermo Scientific). Quantification of *16S* rDNA copies was performed through interpolation from the standard curve with the cycle threshold (Ct) value obtained for each sample. The results are expressed as *16S* rDNA copy/cell.

### Statistical Analysis

Statistical analysis for each experiment was performed between all the conditions, for each timepoint analyzed. Statistical differences were determined using one-way analysis of variance (ANOVA) with a Tukey multiple comparison test. We used GraphPad Prism v6.0 for Windows software (GraphPad Software Inc.) to calculate the mean, the standard error of the mean (SEM), and to perform the statistical tests. A value of p < 0.05 denoted statistical significance.

## Results

### IgM-Beads Favor Lysosomal Acidification in Macrophage-Enriched Cell Cultures Infected by *P. salmonis*

**Figure 1:**
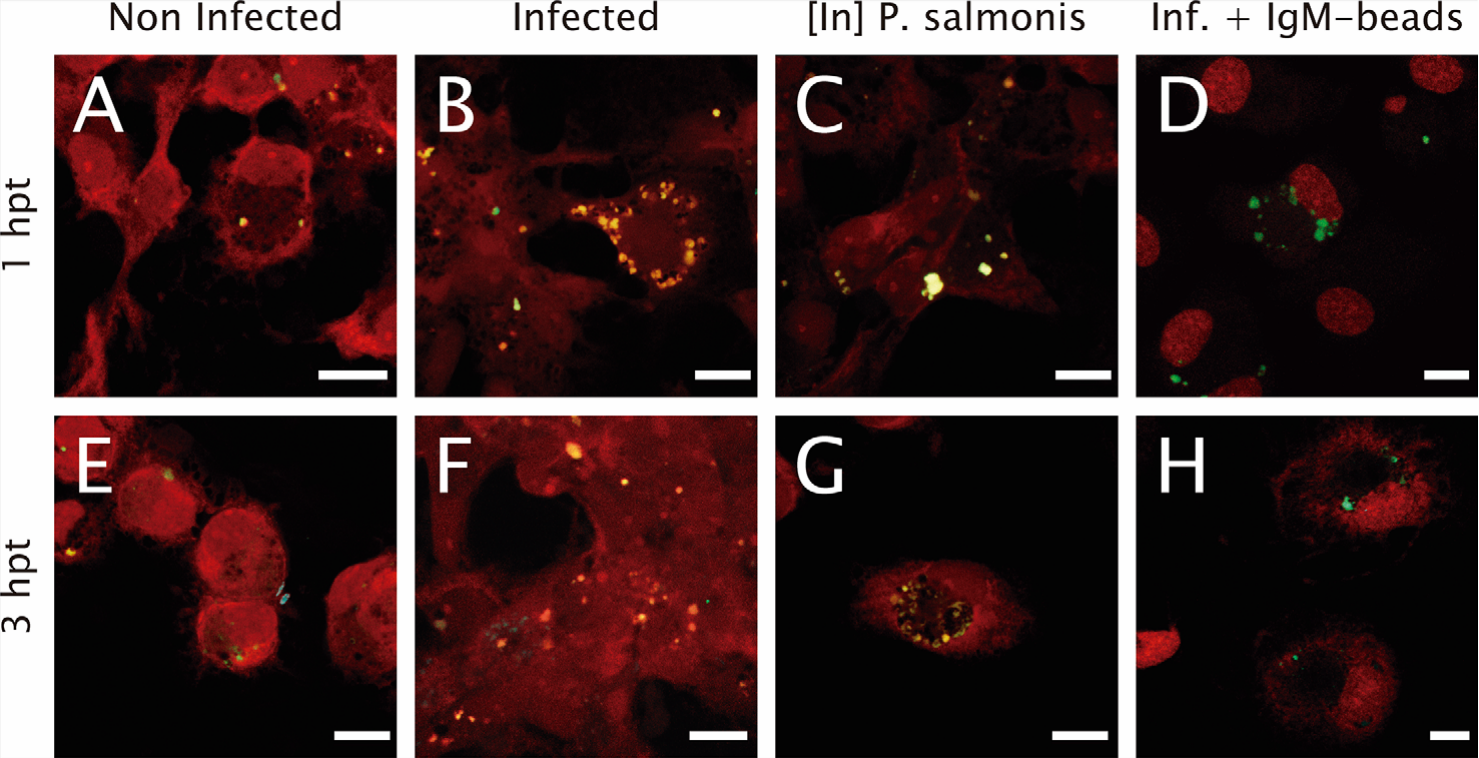
Lysosomal acidification in infected macrophage-enriched cell cultures treated with IgM-beads. Macrophage-enriched cell cultures obtained from Atlantic salmon head kidneys were incubated with *P. salmonis* at a MOI of 10 bacteria/cell. The infected cells were treated with IgM-beads and analyzed at 1 and 3 hpt. The macrophage-enriched cell cultures were treated with the LSYB probe (green and blue) to stain lysosomes and analyze their pH. **(A, E)** Non-infected macrophages analyzed at 1 and 3 hpt. Macrophages-enriched cell cultures infected with *P. salmonis* for 1 hpt **(B)**, and 3 hpt **(F)**. Macrophage-enriched cell cultures incubated with inactivated (In) *P. salmonis* for 1 hpt **(C)** and 3 hpt **(G)**. Macrophage-enriched cultures infected with *P. salmonis* and treated with IgM-beads for 1 hpt **(D)** and 3 hpt **(H)**. Scale bar: 10 μm.

To determine if IgM-beads reverses the modulation of the lysosomal activity induced by bacterial infection, the number of lysosomes and their acidification level were evaluated. In non-infected cells, either 1 or 3 hpt, there is a punctate pattern of yellow/green and cyan fluorescent signal (**Figures 1A, E**). This result suggests the presence of acidic and neutral vesicles along the macrophage-like cell. When we imaged *P. salmonis*-infected macrophage-enriched cell cultures, we also observed at 1 hpt a yellow/green fluorescent punctate pattern (**Figure 1B**). However, only at 3 hpt we found both yellow/green and cyan fluorescent dots (**Figure 1F**), suggesting the presence of acidic and neutral vesicles after treatment. When incubating macrophage-enriched cell cultures with inactivated *P. salmonis*, we observed a pattern of dots of yellow/green fluorescent (**Figures 1C, G**) at both times assayed. This result suggests the presence of acid vesicles generated against inactivated *P. salmonis*. Finally, in macrophage-enriched cells cultures infected with *P. salmonis* and then incubated with IgM-beads (**Figure 1D**), we observed at 1 hpt a punctate pattern of yellow/green fluorescence and at 3 hpt we observed both dots of yellow/green and cyan fluorescence (**Figure 1H**), in a similar way to the observed in infected cells (**Figures 1B, F**).

**Figure 2:**
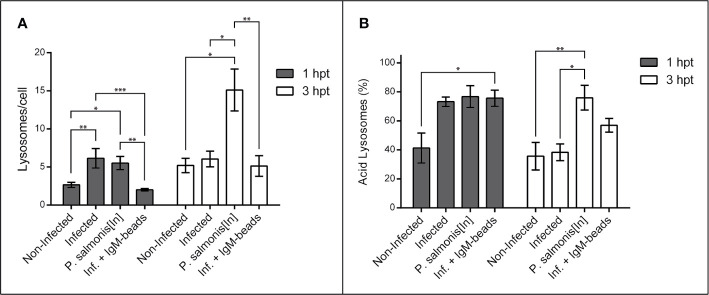
Lysosomal quantification in infected macrophage-enriched cell cultures treated with IgM-beads. Macrophage-enriched cell cultures were infected with *P. salmonis* at a MOI of 10 bacteria/cell and analyzed at 1 and 3 hpt. The lysosomes were stained with the LSYB probe and quantified as acidic lysosomes or neutral-basic (NB) lysosomes. The data were normalized to the number of cells analyzed. **(A)** Total number of lysosomes per cell. **(B)** Percentage of acidic lysosomes for each condition. The statistical analysis was performed through parametric ANOVA with a Tukey multiple comparison test. Significant differences: *p < 0.05, **p < 0.01, ***p < 0.001.

When we quantified the number of lysosomes/cell in macrophage-enriched cell cultures infected with *P. salmonis*, we observed a significant increase in comparison to non-infected cells at 1 hpt. However, this difference was not maintained with longer infection times (**Figure 2A** and **Supplementary Table 2**). When we used inactivated *P. salmonis* during the incubation, we observed a significant increase in comparison to non-infected cells at both times assayed. Nevertheless, the larger effect was found at 3 hpt (**Figure 2A** and **Supplementary Table 2**). On the other hand, when the macrophage-enriched cell cultures infected with *P. salmonis* were incubated with IgM-beads, the results show no difference in the number of lysosomes/cell compared to the non-infected cells (**Figure 2A** and **Supplementary Table 2**). Similarly, when we used the BSA-beads we found no difference compared with the infected macrophage-enriched cell cultures incubated at 1 and 3 hpt (**Supplementary Figure 1A** and **Supplementary Table 2**).

Subsequently, we determined the percentage of acid lysosomes in macrophage-enriched cell cultures. This analysis showed that, despite the slight increase in the number of lysosomes at the first timepoint evaluated, the macrophage-enriched cell cultures infected with *P. salmonis* exhibited a higher percentage of acidic lysosomes in comparison with non-infected cells (**Figure 2B** and **Supplementary Table 3**), although, not significantly different. This increase was even greater in magnitude in macrophage-enriched cell cultures incubated with inactivated *P. salmonis*, and macrophage-enriched cell cultures infected with *P. salmonis* and incubated with IgM-beads at 1 hpt. However, the only group to show significant differences to non-infected cells, were cells infected with *P. salmonis* and incubated with IgM-beads. At longer infection times with *P. salmonis*, the percentage of acid lysosomes decreased by almost half. Nonetheless, the macrophage-enriched cell cultures infected with *P. salmonis* and incubated with IgM-beads at 3 hpt, showed a decrease in the percentage of acid lysosomes, but not enough to reach a statistical difference (**Figure 2B** and **Supplementary Table 3**). Similar percentages of acid lysosomes was observed in macrophage-enriched cell cultures infected with *P. salmonis* and incubated with BSA-beads (**Supplementary Figure 1B** and **Supplementary Table 3**). In contrast, in macrophage-enriched cell cultures incubated with inactivated *P. salmonis*, the percentage of acid lysosomes at both times analyzed was higher than 75% of the total lysosomes in the samples (**Figure 2B** and **Supplementary Table 3**). In summary, the results suggest that the infection of *P. salmonis* prevents the lysosomal acidification, but the effect is attenuated after treatment with protein-beads.

### Incubation With IgM-Beads Reverses the Weak Hydrolytic Activity Induced by *P. salmonis* During Infection of Macrophage-Enriched Cell Cultures

**Figure 3:**
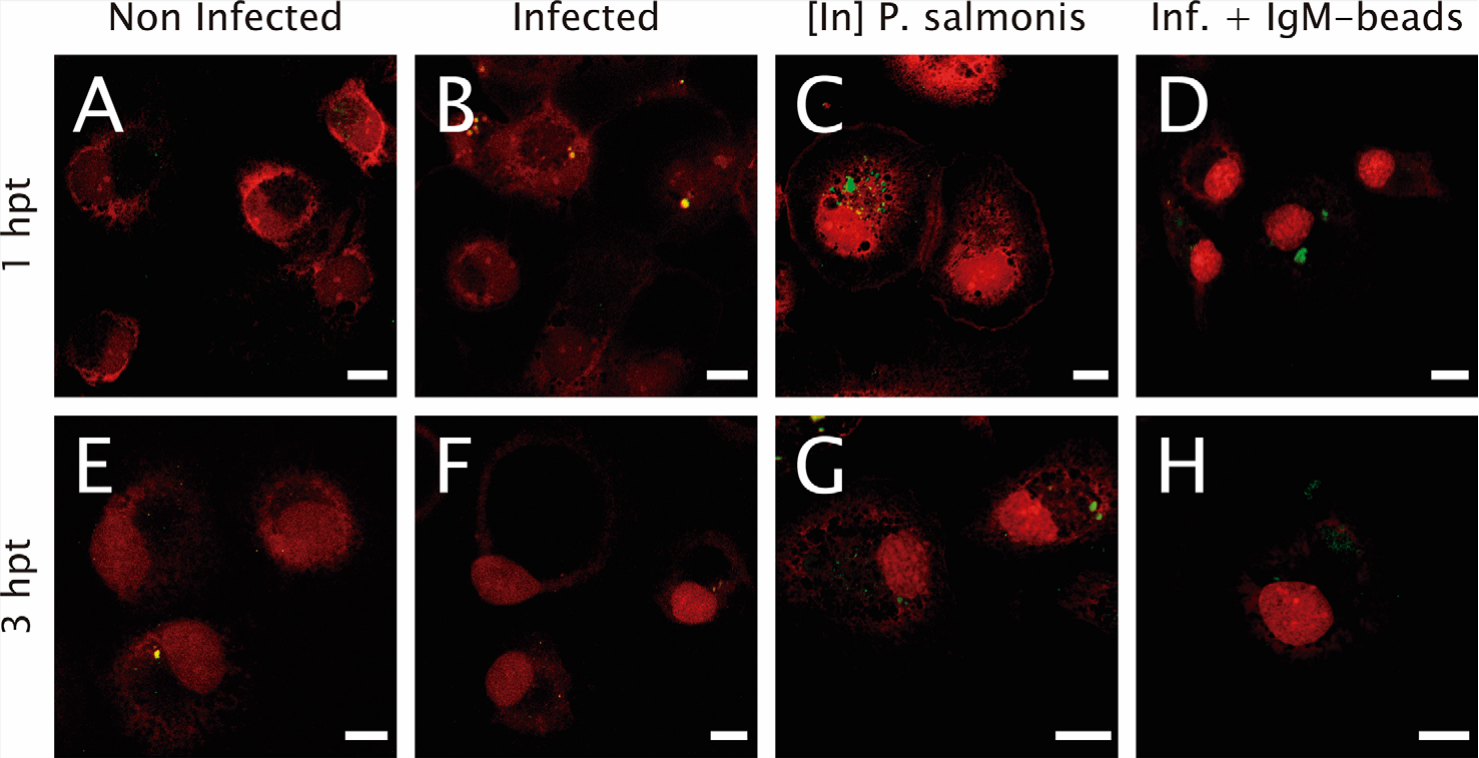
Lysosomal functionality in infected macrophage-enriched cell cultures treated with IgM beads. Macrophage-enriched cell cultures were infected with P. salmonis at a MOI 10 of bacteria/cell, treated with IgM beads and analyzed at 1 and 3 hpt. Cells were treated with the DQ BSA^TM^ Green probe to stain the proteolytic focusses; the nucleus was stained with PI (red). **(A, E)** Non-infected macrophages analyzed at 1 and 3 hpt. Macrophage-enriched cell cultures incubated with P. salmonis for 1 hpt **(B)** and 3 hpt **(F)**. Macrophage-enriched cell cultures incubated with inactivated (In) P. salmonis for 1 hpt **(C)** and 3 hpt **(G)**. Macrophage enriched cell cultures infected with P. salmonis and treated with IgM-beads for 1 hpt **(D)** and 3 hpt **(H)**. Scale bar: 10 μm.

The proteolytic activity was assessed using the fluorescent degrading event marker DQ™ Green BSA. In non-infected macrophage-enriched cell cultures, either 1 or 3 hpt, fluorescent events were evident (**Figures 3A, E**), suggesting proteolytic activity in non-infected cells. Similarly, in infected *P. salmonis* macrophage-enriched cell cultures we observed fluorescent dots due to proteolytic events/cell at both time points evaluated (**Figures 3B, F**). Nevertheless, when we imaged macrophage-enriched cell cultures with inactivated *P. salmonis*, at 1 and 3 hpt, we observed larger clusters with an intense green fluorescent signal due to proteolytic activity at (**Figures 3C, G**). A similar result was observed when IgM-beads were incubated with infected macrophage-enriched cell cultures (**Figures 3D, H**), suggesting that incubation with IgM-beads induced proteolytic activity in infected cells at both times evaluated.

**Figure 4:**
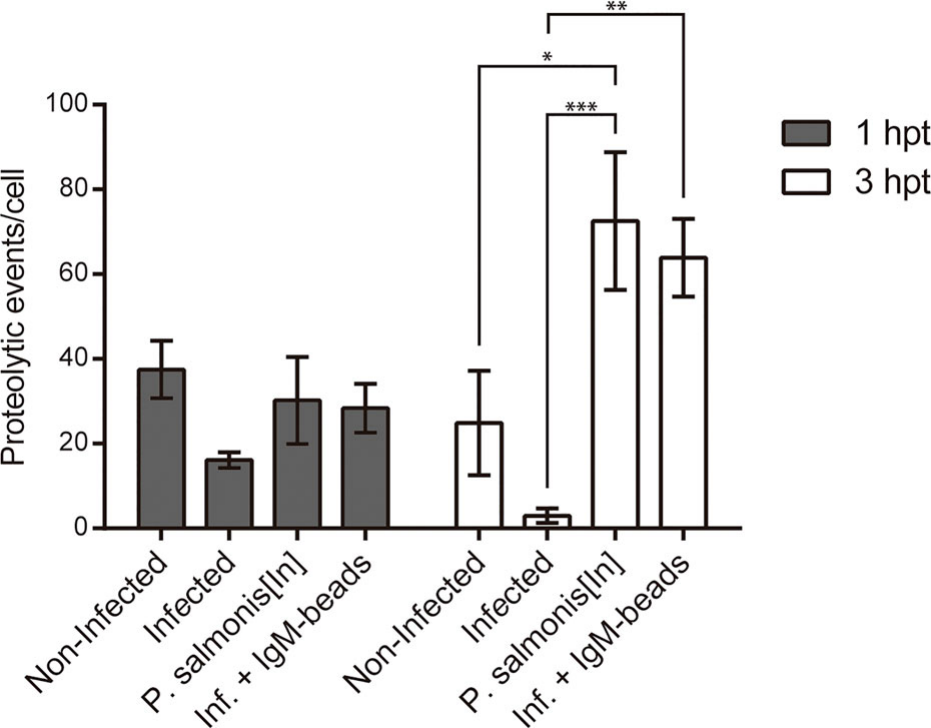
Quantification of proteolytic events in infected macrophage-enriched cell cultures treated with igM-beads. Macrophage-enriched cell cultures were infected with *P. salmonis* at a MOI of 10 bacteria/cell and analyzed at 1 and 3 hpt. The proteolytic events were detected using the DQ-BSA™ Green probe and data were quantified and normalized to the number of cells analyzed for each condition. The statistical analysis was performed through a parametric ANOVA with a Tukey multiple comparison test. Significant differences: *p <0.05, **p < 0.01, ***p<0.001.

In order to determine whether IgM-beads reverse the inhibition of proteolytic activity induced by *P. salmonis* during infection, we quantified the fluorescence events of DQ™ Green BSA degradation using confocal microscopy. At 1 hpt, macrophage-enriched cell cultures infected by *P. salmonis* showed less than half of the proteolytic activity foci compared to non-infected cells. Despite this decrease in activity, statistical analysis suggested non-significant differences between both conditions. In the case of macrophage-enriched cell cultures infected with *P. salmonis* and incubated with IgM-beads, the proteolytic foci were similar to those observed in non-infected cells and in macrophage-enriched cell cultures incubated with inactivated *P. salmonis* (**Figure 4** and **Supplementary Table 4**).

At the second timepoint evaluated (3 hpt), the number of proteolytic foci observed in macrophage-enriched cell cultures infected by *P. salmonis* was significantly lower in comparison to proteolytic events/cell observed in infected cells incubated with IgM-beads. This induction is similar to that observed in macrophage-enriched cell cultures incubated with inactivated *P. salmonis* (**Figure 4** and **Supplementary Table 4**). The increase in proteolytic foci was not observed in the infected macrophage-enriched cell cultures incubated with BSA-beads, at either timepoint analyzed (**Supplementary Figure 2** and **Supplementary Table 4**). Collectively, these results suggest that IgM-beads reversed the inhibition of lysosomal hydrolytic activity induced by *P. salmonis* during infection of macrophage-enriched cell cultures derived from Atlantic salmon head kidney.

### IgM-Beads Decrease the Cytotoxicity in Macrophage-Enriched Cell Cultures Infected by *P. salmonis*

**Figure 5:**
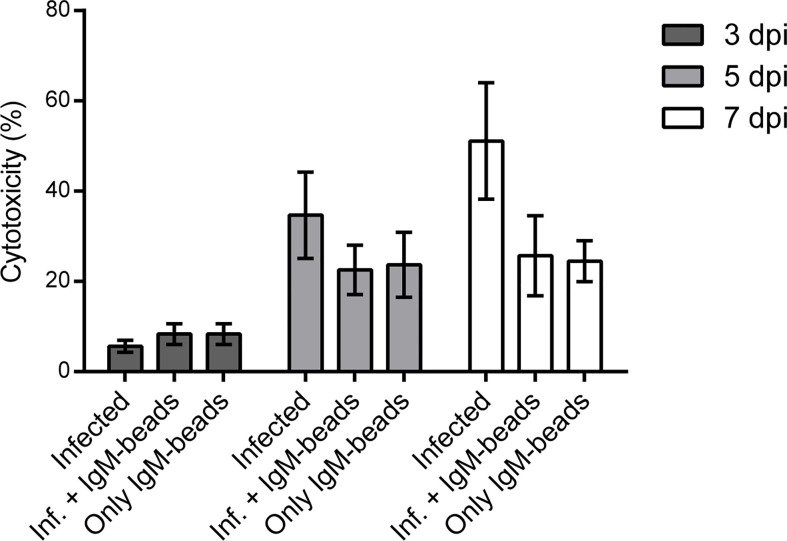
Evaluation of cytotoxicity induced by IgM-beads. Macrophage-enriched cell cultures were infected with *P. salmonis* at MOI of 10 bacteria/cell and treated with IgM-beads. The cytotoxicity was evaluated at 3, 5, and 7 dpi by the detection LDH release into the extracellular medium. The statistic test was performed using ANOVA with a Tukey multiple comparison test, the analysis was performed individually for each evaluated time.

We used the LDH assay as a reporter for cell death to evaluate the cytotoxicity induced by *P. salmonis* when it infects macrophage-enriched cell cultures and the possible effect that the IgM-beads triggers. The cytotoxicity induced by *P. salmonis* in infecting macrophage-enriched cell cultures 3 dpi, was similar to that observed in infected cells incubated with IgM-beads (**Figure 5**, left panel, and **Supplementary Table 5**), although not significantly different. In the same way, a similar cytotoxicity was observed in macrophage-enriched cell cultures incubated with only IgM-beads. At 5 dpi, the cytotoxicity induced by *P. salmonis* was higher than the effect obtained with *P. salmonis* infection and IgM-beads. However, no significant differences were observed between both conditions. As control, the cytotoxicity induced by incubation with only IgM-beads was similar to the infected group, but neither reach significant differences with the other conditions evaluated (**Figure 5**, middle panel, and **Supplementary Table 5**). At 7 dpi, the higher cytotoxicity was induced by *P. salmonis*, which was higher than in macrophage-enriched cell cultures infected by *P. salmonis* and incubated with IgM-beads. However, despite that the treatment with IgM-beads produces half the level of cytotoxicity to that observed in infected cells, although there is no significant difference between both conditions. Similarly, the cytotoxicity induced by the incubation with only IgM-beads reached ~25% (**Figure 5**, right panel, and **Supplementary Table 5**). Additionally, we evaluated the effect of BSA-beads on the cytotoxicity induced by *P. salmonis* during infection of macrophage-enriched cell cultures, and we observed that it was similar to the cells infected by *P. salmonis* at 5 and 7 dpi (**Supplementary Figure 3** and **Supplementary Table 5**). These results suggest that the effect of IgM-beads on the cytotoxicity of macrophage-enriched cell cultures is due to the presence of IgM.

### IgM-Beads Promote Bacterial Clearance in Macrophage-Enriched Cell Cultures Infected by *P. salmonis*

**Figure 6:**
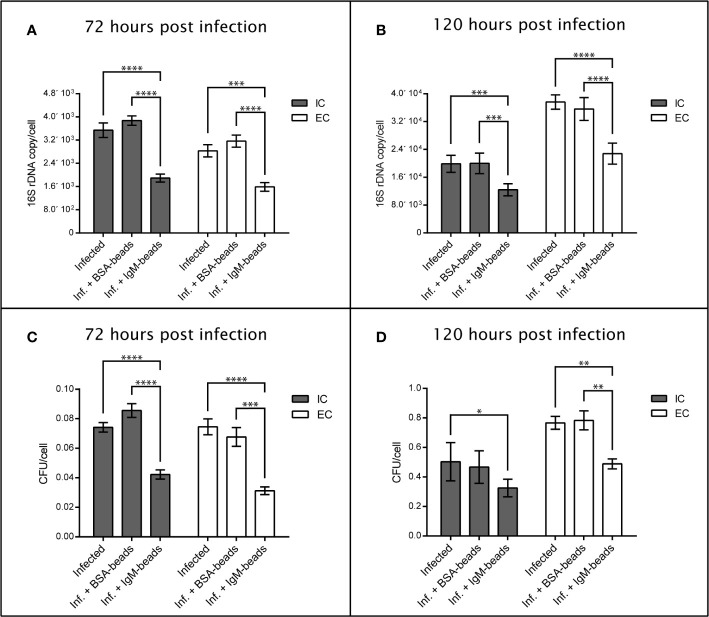
Quantification of extracellular and intracellular P. salmonis recovered from SHK-1 infected cells treated with IgM-beads. SHK-1 cells were infected with P. salmonis at a MOI of 10 bacteria/cell and treated with IgM-beads. SHK-1 cells incubated with BSA-beads were used as controls for each time point. The bacterial load was determined by quantification of 16S rDNA copy/cell at 72 hpi **(A)** and 120 hpi **(B)**, and also by quantification of CFU/cell at 72 hpi **(C)** and 120 hpi **(D)**. The statistical analysis was performed through a parametric ANOVA with a Tukey multiple comparison test. Significant differences: *p < 0.05, **p < 0.01, ***p < 0.001, ****p < 0.0001.

To evaluate the effect of IgM-beads on the survival and replication of *P. salmonis* when it infected Atlantic salmon macrophages, we used the SHK-1 cell line, which is derived from leukocytes and possesses the properties of macrophages (40). The bacterial load was evaluated by quantification of *16S* rDNA copies/cell and CFU/cell. In both cases, quantification was performed from the supernatant and intracellular medium of *P. salmonis*-incubated macrophages. At 72 hpi the incubation of SHK-1 cells infected by *P. salmonis* with IgM-beads resulted in a significant decrease in about 50% of the number of copies of *16S* rDNA/cell, in comparison with that in infected cells that were not incubated with IgM-beads (**Figure 6A** and **Supplementary Table 6**). As control, infected cells were incubated with BSA-beads, which have no effect over the number of copies of *16S* rDNA/cell (**Figure 6A** and **Supplementary Table 6**). A similar result was observed at 120 hpi, incubation of infected SHK-1 cells with IgM-beads resulting in a significant decrease of bacterial load, both intracellular and extracellular, in comparison with that observed in infected cells that had not been treated with IgM-beads. As control, infected SHK-1 cells were incubated with BSA-beads, although no effect were observed on bacterial load (**Figure 6B** and **Supplementary Table 6**).

On the other hand, the effects of IgM-beads on bacterial load were evaluated by recovery of CFU present inside as well as outside the SHK-1 cells. We observed that, at 72 and 120 hpi, incubation of IgM-beads in infected SHK-1 cells induced a significant decrease in recovered CFU both intracellularly as well as in the infection supernatant, in comparison with SHK-1 cells infected by *P. salmonis* (**Figures 6C, D** and **Supplementary Table 6**). As control, CFU/cell were quantified from intracellular medium and infection supernatant of infected SHK-1 cells incubated with BSA-beads, however these protein beads control do not decrease the bacterial load (**Figures 6C, D** and **Supplementary Table 6**).

Together, these results suggest that incubation with IgM-beads affects the viability and replication of *P. salmonis* when it infects macrophage-like cells. This effect was not observed when we utilized BSA-beads, in all cases evaluated (**Figure 6** and **Supplementary Table 6**).

## Discussion

Antibody-based therapies, which have been widely used as a passive immunization strategy against various pathogens (19, 20, 41–44), are capable of stimulating the endocytic pathway through the interaction between Fc and FcR (45). In mammals, this binding activates a phosphoinositol 3-kinase (PI3-K) protein-dependent signal and downstream signaling pathways that lead to a wide variety of effector mechanisms including oxidative burst, increased phagocytosis, cytokine release, and increased antigen presentation and routing. In addition, the Fc-FcR interaction is responsible for routing the vesicular traffic that is altered by intracellular pathogens, which allows the bacteria to undergo degradation in the lysosome (19, 20, 46).

Antibody-based therapies using beads coated with immunoglobulins have been previously reported in treatment against *L. pneumophila* and *Streptococcus dysgalactiae*; these treatments stimulate the intracellular response by inducing bacterial clearance (19, 42). IgG-beads against *L. pneumophila* infection stimulate the intracellular response of murine macrophages (19). These IgG-beads induce host cells to become non-permissive for intracellular replication; as a result, intracellular pathogens are targeted to the lysosome for degradation. The use of IgY-beads against *S. dysgalactiae*, the causal agent of cow mastitis, have been demonstrated to be effective in inhibiting propagation and inducing the phagocytosis of bacteria (42). In fish, although stimulation of the endocytic pathway through the Fc-FcR interaction has not been described to date, passive immunization strategies based on the intraperitoneal delivery of specific antibodies against bacteria such as *Flavobacterium psychrophilum*, *Yersinia ruckerii*, and *Vibrio anguillarum* have been reported in rainbow trout. In all of these strategies, a reduction in the mortality and aggressiveness index of infectious outbreaks was observed (27, 28, 47), suggesting that these passive immunization strategies are capable of stimulating a protective response in fish.

Recently, it has been described that *P. salmonis* is able to persist in macrophage-enriched cell cultures for at least 120 hpi, where it evades the host response by reducing lysosomal acidification and its proteolytic activity in the first hpi (16). In the current work, we evaluated the functionality of this organelle by assessing lysosomal acidity and proteolytic activity of macrophages infected with *P. salmonis* and stimulated with IgM-beads. Results showed that incubation of infected cells with IgM-beads promotes an increase in the acidity of lysosomes as well as an increase of about 2 folds in the number of proteolytic foci per cell, in comparison with cells infected with *P. salmonis* that were not incubated with IgM-beads. In the latter, the pH was maintained in a neutral–basic range similar to that observed in non-infected macrophages.

The mechanisms of cell death induced by *P. salmonis* are poorly described. However, it is reported that in early, intermediate, and late stages of infection of *P. salmonis* in trout monocytes/macrophages like cells, apoptosis was induced in about 22–30% of infected cells; this represents a mechanism of *P. salmonis* to promote apoptosis in a fraction of phagocytic cells. Rojas et al. (48) suggested that apoptosis may allow the majority of the macrophage population to be productively infected by the bacterium, and ensure the initiation of bacterial infection and subsequent spread to other tissues (48). Furthermore, *P. salmonis* is able to infect various salmonid cell lines, such as CHSE-214, RTS-11, RTG-2, ASK, and SHK-1 (4, 14, 31, 49–52). In all of them, *P. salmonis* induces a CPE characterized by the production of clusters of rounded and vacuolated cells, which culminated with the detachment of the monolayer and delayed cell lysis (49, 51, 52). In our results, we observed that infection by *P. salmonis* induced cell lysis in about 50% of infected cells after 7 dpi. Similar results were reported by Oliver et al. (53), who reported cell lysis in about 60% of infected cells after 8 dpi and also by Hernández et al. (54), who observed cell lysis in almost 60% of infected cells after 9 dpi. In our work, we use the same strategy implemented by Oliver et al. (53) and Hernandez et al. (54), where cell lysis was determined by the quantification of LDH leakage (53, 54). The enzyme activity is an indicator of irreversible damage to the plasma membrane and the inability of cells to retain intracellular enzymes (55). Together, these results suggest that a fraction of the infected macrophages can be driven to apoptosis, while other fraction eventually could evolve to necrosis since both routes of cell death can be independently regulated. These parallel running pathways are the case of infection of alveolar macrophages by *Mycobacterium tuberculosis*, where bacteria can control cell death pathways: apoptotic cell death is bactericidal, whereas necrotic cell death may facilitate bacterial dissemination and transmission (56). However, the mechanisms of host cell death in Atlantic salmon macrophages infected by *P. salmonis* are not well understood and require further studies.

Recently, Oliver et al. (53) described a protective effect of both IgY antibody against a whole protein extract of *P. salmonis*, as also IgY antibody against *P. salmonis* Hsp60, when SHK-1 cells are incubated with these antibodies previous to be infected with *P. salmonis*, observing a cell lysis similar to non-infected cells (~20%) at 8 dpi. Interestingly, in our work the incubation of infected cells with IgM-beads decreased cell lysis to 25% of total cells, suggesting a protective effect of incubation with non-specific antibodies. This effect was not observed when infected macrophage-enriched cell cultures were incubated with BSA-beads, suggesting that, in a similar way to that in the work of Joller et al. (19), this protection would involve FcR signaling. However, further studies are needed to validate this hypothesis.

The classical treatment of pathogenic infection by passive immunization includes the use of soluble antibodies, which bind to the pathogen and promote its phagocytosis and lysis by the complement pathway. However, the utilization of a carrier loaded with antibodies induces phagocytosis of macrophages and neutrophils *via* FcR. Thus, increasing bacterial uptake by phagocytes is expected to contribute to reduce the inflammation (42). Confirmation that IgM-beads induce protection against infection with *P. salmonis* was obtained by analysis of bacterial load in infected cells. After stimulating macrophage-like cells infected by *P. salmonis* with IgM-beads, we observed a reduction in bacterial load after 72 and 120 hpi. This was evidenced by quantifying the bacterial load in the extracellular medium and inside the cell through the determination of the number of copies of *16S* rDNA gene by qPCR and CFU counting. These results are similar to those obtained by Joller et al. (19), where the authors described a decrease in the viable bacterial load after incubating murine macrophages infected by *L. pneumophila* with IgG-beads. Joller et al. (19) proposed that treatment with IgG-beads mediates host cell protection against intracellular pathogens that reside in vacuoles and evade phagolysosomal fusion, by re-routing the pathogens to undergo lysosomal degradation.

The quantification of bacterial load recovered in agar CHAB was inconsistent with the number of *P. salmonis* utilized to infect SHK-1 cells (10 bacteria for each cell). This discrepancy is probably due to that *P. salmonis* is considered be highly fastidious—even previously was considered to be cultivable only in eukaryotic cell lines—than may be cultured on cell-free agar, but with demanding nutritional requirements which could hinder the growth of recovered bacteria, according to the facultative intracellular nature of this fish pathogen (2, 57). Similar results were observed in Yañez et al. (50), where the authors described that efficiency of recovering of *P. salmonis* in cell-free agar media is not suitable to determine the CFU concentration, mainly because the bacterial growth is extremely low.

Intracellular pathogens invade their hosts *via* a cell entry process that culminates in the formation of a pathogen-containing vacuoles, where it replicates and prevents their fusion with lysosomes. This evasion requires to modulation of the membrane Rab proteins (58). Rab proteins are small GTPases of the endocytic pathway that participate in the maturation of the endosome prior to fusion and delivery of their contents for lysosomal degradation, where Rab4 and Rab5 proteins are characteristic of early endosomes and Rab7 is indicative to late endosomes (59). In fish, the presence of 52 Rab GTPases have been described in channel catfish (60), and have also been described in the cellular distribution and transcriptional regulation of Rab5c and Rab7a in CHSE-214 cells, where both have the same function as those described in mammals (61). In our work, we have observed that IgM-beads promotes the lysosomal activity in infected macrophage-enriched cell cultures, as well as decreasing the bacterial load, suggesting that the IgM-beads effect over the macrophage-enriched cells could be mediated by reversing the lysosomal evasion from *P. salmonis*, opening an interesting question to further research about the mechanism promoted by IgM-beads over the exchange of Rab proteins.

In this *in vitro* approach, we observed that treatment with IgM-beads resulted in a decrease in the bacterial load. However, the mechanism by which this occurs is not yet understood, because it is necessary to determine whether IgM-beads can lead *P. salmonis* to cause lysosomal degradation, as is the case against *L. pneumophila*, another intracellular pathogen. Although our results suggest this to be the case, but further verification of this is required.

## Data Availability Statement

The original contributions presented in the study are included in the article/**Supplementary Material**. Further inquiries can be directed to the corresponding author.

## Ethics Statement

The animal study was reviewed and approved by Institutional Ethics Committee of Universidad de Santiago de Chile.

## Author Contributions

DP-S, AE, and ST performed most of the experiments, analyzed data, and prepared figures. JM-R and CB performed any experiments. EV-V, DT-A, JR-P, AS, and ES contributed to review the manuscript. SR-C conceived and supervised the study. DP-S, FR-L, JR-P, and SR-C wrote the manuscript. All authors contributed to the article and approved the submitted version.

## Funding

This work was financially supported by FONDECYT Iniciación grant 11150807 (SR-C), 11180621 (DT-A), and 11180705 (JR-P), Start-Up UMayor 101205 (SR-C), CORFO 13CTI-21527, FDP UMayor PEP-I 2019082 (SR-C), and PCI-ANID REDES180097 (SR-C). The authors also acknowledge the fellowship support DYCIT USACH 041831MH-Postdoc (JR-P) and 022043IB-Postdoc (EV-V), National Agency for Research and Development (ANID)/Scholarship Program/DOCTORADO BECAS CHILE/2019 – 21191135 fellowship to DP-S, UMayor-Ph.D. fellowships to DP-S.

## Conflict of Interest

ST, JM-R, CB, AS and ES were employed by Ictio Biotechnologies S.A.

The remaining authors declare that the research was conducted in the absence of any commercial or financial relationships that could be construed as a potential conflict of interest.

## Supplementary Material

Supplementary Figure 1 Lysosomal quantification in infected macrophage-enriched cell cultures treated with IgM-beads and BSA-beads. Macrophage-enriched cell cultures were infected with *P. salmonis* at MOI of 10 bacteria/cell and analyzed at 1 and 3 hpt. The lysosomes were stained with the LSYB probe and quantified as acidic lysosomes or neutral-basic (NB) lysosomes. The data were normalized to the number of cells analyzed. **(A)** Total number of lysosomes per cell. **(B)** Percentage of acidic lysosomes for each condition. The statistical analysis was performed through parametric ANOVA with a Tukey multiple comparison test. Significant differences: *p < 0.05.

Supplementary Figure 2 Quantification of proteolytic events in infected macrophage-enriched cell cultures treated with IgM-beads and BSA-beads. Macrophage-enriched cell cultures were infected with *P. salmonis* at MOI of 10 bacteria/cell and analyzed at 1 and 3 hpt. The proteolytic events were detected using the DQ-BSA^TM^ Green probe and data were quantified and normalized to the number of cells analyzed for each condition. The statistical analysis was performed through a parametric ANOVA with a Tukey multiple comparison test. Significant differences: *p <0.05, **p < 0.01.

Supplementary Figure 3 Evaluation of cytotoxicity in infected macrophage-enriched cell cultures treated with IgM-beads and BSA-beads. Macrophage-enriched cell cultures obtained from *S. salar* were infected with *P. salmonis* at MOI of 10 bacteria/cell and treated with IgM-beads. The cytotoxicity was evaluated at 3, 5 and 7 dpi by the detection of LDH release into the extracellular medium. The statistical analysis was performed through a parametric ANOVA with a Tukey multiple comparison test. Significant differences: *p <0.05, **p < 0.01, ***p<0.001, **** p<0.0001.

## References

[1] Fryer JL Hedrick RP Piscirickettsia salmonis: a Gram-negative intracellular bacterial pathogen of fish. J Fish Dis (2003) 26(5):251–62. 10.1046/j.1365-2761.2003.00460.x 12962234

[2] Rozas M Enriquez R Piscirickettsiosis and Piscirickettsia salmonis in fish: a review. J Fish Dis (2014) 37(3):163–88. 10.1111/jfd.12211 24279295

[3] Branson EJ Nieto-Diaz Muñoz D Description of a new disease condition occurring in farmed coho salmon, Oncorhynchus kisutch (Walbaum), in South America. J Fish Dis (1991) 14:147–56. 10.1111/j.1365-2761.1991.tb00585.x

[4] Cvitanich JD O. Garate N Smith CE The isolation of a rickettsial-like organism causing disease and mortality in Chilean salmonids and its confirmation by Koch’s postulate. J Fish Dis (1991) 14:121–45. 10.1111/j.1365-2761.1991.tb00584.x

[5] Aqua Sernapesca: “El SRS sigue siendo el mayor problema sanitario que enfrenta la salmoniculttura”. Editorial group Editec SpA (2012). Available at: http://www.aqua.cl/2012/11/23/sernapesca-el-srs-sigue-siendo-el-mayor-problema-sanitario-que-enfrenta-la-salmoniculttura/#.

[6] SERNAPESCA Informe Sanitario Acuícola año 2017. Chile: Departamento de Salud Animal, Subdirección de Acuicultura, Servicio Nacional de Pesca y Acuicultura (2018).

[7] Marshall SH Conejeros P Zahr M Olivares J Gómez FA Cataldo P Immunological characterization of a bacterial protein isolated from salmonid fish naturally infected with Piscirickettsia salmonis. Vaccine (2007) 25(11):2095–102. 10.1016/j.vaccine.2006.11.035 17250933

[8] SERNAPESCA Informe Sanitario Acuícola año 2012, Unidad de Salud Animal, Subdirección de Acuicultura, Servicio Nacional de Pesca y Acuicultura. Chile: Servicio Nacional de Pesca y Acuicultura, Gobierno de Chile (2013).

[9] SERNAPESCA Informe Sanitario Acuícola año 2018. Chile: Departamento de Salud Animal, Subdirección de Acuicultura, Servicio Nacional de Pesca y Acuicultura (2019).

[10] SERNAPESCA Informe sobre uso de antimicrobianos en la salmonicultura nacional. In: Animal D, editor. Sudirección de Acuicultura. Valparaíso: National Fishing Service dependent on the Ministry of Economy, Development and Tourism of the Government of Chile (2019).

[11] Maisey K Montero R Christodoulides M Vaccines for piscirickettsiosis (salmonid rickettsial septicaemia, SRS): the Chile perspective. Expert Rev Vaccines (2017) 16(3):215–28. 10.1080/14760584.2017.1244483 27690686

[12] Figueroa J Castro D Lagos F Cartes C Isla A Yáñez AJ Analysis of single nucleotide polymorphisms (SNPs) associated with antibiotic resistance genes in Chilean Piscirickettsia salmonis strains. J Fish Dis (2019) 42(12):1645–55. 10.1111/jfd.13089 31591746

[13] Gomez FA Tobar JA Henríquez V Sola M Altamirano C Marshall SH Evidence of the presence of a functional Dot/Icm type IV-B secretion system in the fish bacterial pathogen Piscirickettsia salmonis. PloS One (2013) 8(1):e54934. 10.1371/journal.pone.0054934 23383004 PMC3557282

[14] Rojas V Galanti N Bols NC Marshall SH Productive infection of Piscirickettsia salmonis in macrophages and monocyte-like cells from rainbow trout, a possible survival strategy. J Cell Biochem (2009) 108(3):631–7. 10.1002/jcb.22295 19681041

[15] McCarthy UM Bron JE Brown L Pourahmad F Bricknell IR Thompson KD Survival and replication of Piscirickettsia salmonis in rainbow trout head kidney macrophages. Fish Shellfish Immunol (2008) 25(5):477–84. 10.1016/j.fsi.2008.07.005 18691656

[16] Perez-Stuardo D Morales-Reyes J Tapia S Ahumada DE Espinoza A Soto-Herrera V Non-lysosomal Activation in Macrophages of Atlantic Salmon (Salmo salar) After Infection With Piscirickettsia salmonis. Front Immunol (2019) 10:434. 10.3389/fimmu.2019.00434 30941123 PMC6433878

[17] Álvarez CA Gomez FA Mercado L Ramírez R Marshall SH Piscirickettsia salmonis Imbalances the Innate Immune Response to Succeed in a Productive Infection in a Salmonid Cell Line Model. PloS One (2016) 11(10):e0163943. 10.1371/journal.pone.0163943 27723816 PMC5056700

[18] Roig J Rello J Legionnaires’ disease: a rational approach to therapy. J Antimicrob Chemother (2003) 51(5):1119–29. 10.1093/jac/dkg191 12668578

[19] Joller N Weber SS Müller AJ Spörri R Selchow P Sander P Antibodies protect against intracellular bacteria by Fc receptor-mediated lysosomal targeting. Proc Natl Acad Sci U S A (2010) 107(47):20441–6. 10.1073/pnas.1013827107 PMC2996673 21048081

[20] Joller N Weber SS Oxenius A Antibody-Fc receptor interactions in protection against intracellular pathogens. Eur J Immunol (2011) 41(4):889–97. 10.1002/eji.201041340 21413006

[21] Griffin BR Opsonic effect of rainbow trout (Salmo gairdneri) antibody on phagocytosis of Yersinia ruckeri by trout leukocytes. Dev Comp Immunol (1983) 7(2):253–9. 10.1016/0145-305X(83)90006-X 6873424

[22] Kodama H Yamada F Murai T Nakanishi Y Mikami T Izawa H Response of rainbow trout immunized with formalin-killed Vibrio anguillarum: Activity of phagocytosis of fish macrophages and opsonising effect of antibody. Fish Pathol (1985) 20(2/3):395–402. 10.3147/jsfp.20.395

[23] Honda A Kodama H Moustafa M Yamada F Mikami T Izawa H Phagocytic activity of macrophages of rainbow trout against Vibrio anguillarum and the opsonising effect of antibody and complement. Res Vet Sci (1986) 40(3):328–32. 10.1016/S0034-5288(18)30544-7 3738229

[24] Sakai DK Opsonization by fish antibody and complement in the immune phagocytosis by peritoneal exudate cells isolated from salmonid fishes. J Fish Dis (1984) 7(1):29–38. 10.1111/j.1365-2761.1984.tb00904.x

[25] O’Dowd AM Ellis AE Secombes CJ Binding of soluble immune complexes to fractionated Atlantic salmon (Salmo salar L.) leucocytes. Vet Immunol Immunopathol (1999) 68(2-4):149–57. 10.1016/S0165-2427(99)00018-5 10438315

[26] O’Dowd AM Ellis AE Secombes CJ Binding of immune complexes to Atlantic salmon peripheral blood leucocytes. Dev Comp Immunol (1998) 22(4):439–48. 10.1016/S0145-305X(98)00018-4 9699489

[27] Arasteh N Aminirissehei A-H Yousif AN Albright LJ Durance TD Passive immunization of rainbow trout (Oncorhynchus mykiss) with chicken egg yolk immunoglobulins (IgY). Aquaculture (2004) 231(1-4):23–36. 10.1016/j.aquaculture.2003.11.004

[28] LaFrentz BR LaPatra SE Jones GR Cain KD Passive immunization of rainbow trout, Oncorhynchus mykiss (Walbaum), against Flavobacterium psychrophilum, the causative agent of bacterial coldwater disease and rainbow trout fry syndrome. J Fish Dis (2003) 26(7):371–84. 10.1046/j.1365-2761.2003.00468.x 12946006

[29] Braun-Nesje R Bertheussen K Kaplan G Seljelid R Salmonid macrophages: separation, in vitro culture and characterization. J Fish Dis (1981) 4:141–51. 10.1111/j.1365-2761.1981.tb01118.x

[30] Fryer JL Lannan CN Giovannoni SJ Wood ND Piscirickettsia salmonis gen. nov., sp. nov., the causative agent of an epizootic disease in salmonid fishes. Int J Syst Bacteriol (1992) 42(1):120–6. 10.1099/00207713-42-1-120 1371057

[31] Henriquez M González E Marshall SH Henríquez V Gómez FA Martínez I A novel liquid medium for the efficient growth of the salmonid pathogen Piscirickettsia salmonis and optimization of culture conditions. PloS One (2013) 8(9):e71830. 10.1371/journal.pone.0071830 24039723 PMC3764132

[32] Wang MX Cheng XY Jin M Cao YL Yang YP Wang JD TNF compromises lysosome acidification and reduces alpha-synuclein degradation via autophagy in dopaminergic cells. Exp Neurol (2015) 271:112–21. 10.1016/j.expneurol.2015.05.008 26001614

[33] DeLuca D Wilson M Warr GW Lymphocyte heterogeneity in the trout, Salmo gairdneri, defined with monoclonal antibodies to IgM. Eur J Immunol (1983) 13(7):546–51. 10.1002/eji.1830130706 6347695

[34] Schindelin J Arganda-Carreras I Frise E Kaynig V Longair M Pietzsch T Fiji: an open-source platform for biological-image analysis. Nat Methods (2012) 9(7):676–82. 10.1038/nmeth.2019 PMC3855844 22743772

[35] Marwaha R Sharma M DQ-Red BSA Trafficking Assay in Cultured Cells to Assess Cargo Delivery to Lysosomes. Bio Protoc (2017) 7(19):e2571. 10.21769/BioProtoc.2571 PMC5657473 29082291

[36] Frost LS Dhingra A Reyes-Reveles J Boesze-Battaglia K The Use of DQ-BSA to Monitor the Turnover of Autophagy-Associated Cargo. Methods Enzymol (2017) 587:43–54. 10.1016/bs.mie.2016.09.052 28253971 PMC5338641

[37] Russell DG Vanderven BC Glennie S Mwandumba H Heyderman RS The macrophage marches on its phagosome: dynamic assays of phagosome function. Nat Rev Immunol (2009) 9(8):594–600. 10.1038/nri2591 19590530 PMC2776640

[38] Mikalsen J Skjaervik O Wiik-Nielsen J Wasmuth MA Colquhoun DJ Agar culture of Piscirickettsia salmonis, a serious pathogen of farmed salmonid and marine fish. FEMS Microbiol Lett (2008) 278(1):43–7. 10.1111/j.1574-6968.2007.00977.x 18028392

[39] Karatas S Mikalsen J Steinum TM Taksdal T Bordevik M Colquhoun DJ Real time PCR detection of Piscirickettsia salmonis from formalin-fixed paraffin-embedded tissues. J Fish Dis (2008) 31(10):747–53. 10.1111/j.1365-2761.2008.00948.x 18681901

[40] Dannevig BH Brudeseth BE GjØen T Rode M Wergeland HI Evensen Ø Characterisation of a long-term cell line (SHK-1) developed from the head kidney of Atlantic salmon (Salmo salar L.). Fish Shellffish Immunol (1997) 7:213–26. 10.1006/fsim.1996.0076

[41] Casadevall A Scharff MD Serum therapy revisited: animal models of infection and development of passive antibody therapy. Antimicrob Agents Chemother (1994) 38(8):1695–702. 10.1128/AAC.38.8.1695 PMC284624 7985997

[42] Aizenshtein E Pinchasov Y Morag E Leitner G Shpanir Y Reimond D Immunological complex for enhancement of innate immune response in passive vaccination. Vaccine (2013) 31(4):626–31. 10.1016/j.vaccine.2012.11.058 23212028

[43] Herrada AA Contreras FJ Tobar JA Pacheco R Kalergis AM Immune complex-induced enhancement of bacterial antigen presentation requires Fcgamma receptor III expression on dendritic cells. Proc Natl Acad Sci U S A (2007) 104(33):13402–7. 10.1073/pnas.0700999104 PMC1948949 17679697

[44] Casadevall A Dadachova E Pirofski LA Passive antibody therapy for infectious diseases. Nat Rev Microbiol (2004) 2(9):695–703. 10.1038/nrmicro974 15372080

[45] Nimmerjahn F Ravetch JV Antibody-mediated modulation of immune responses. Immunol Rev (2010) 236:265–75. 10.1111/j.1600-065X.2010.00910.x 20636822

[46] Pincetic A Bournazos S DiLillo DJ Maamary J Wang TT Dahan R Type I and type II Fc receptors regulate innate and adaptive immunity. Nat Immunol (2014) 15(8):707–16. 10.1038/ni.2939 PMC7430760 25045879

[47] Lee SB Mine Y Stevenson RM Effects of hen egg yolk immunoglobulin in passive protection of rainbow trout against Yersinia ruckeri. J Agric Food Chem (2000) 48(1):110–5. 10.1021/jf9906073 10637061

[48] Rojas V Galanti N Bols NC Jiménez V Paredes R Marshall SH Piscirickettsia salmonis induces apoptosis in macrophages and monocyte-like cells from rainbow trout. J Cell Biochem (2010) 110(2):468–76. 10.1002/jcb.22560 20432244

[49] Fryer JL Lannan CN Garcés LH Larenas JJ Smith PA Isolation of a Rickettsiales-Like Organism from Diseased Coho Salmon (Oncorhynchus kisutch)in Chile. Fish Pathol (1990) 25(2):107–14. 10.3147/jsfp.25.107

[50] Yanez AJ Valenzuela K Silva H Retamales J Romero A Enriquez R Broth medium for the successful culture of the fish pathogen Piscirickettsia salmonis. Dis Aquat Organ (2012) 97(3):197–205. 10.3354/dao02403 22422090

[51] Smith PA Díaz FE Rojas ME Díaz S Galleguillos M Carbonero A Effect of Piscirickettsia salmonis inoculation on the ASK continuous cell line. J Fish Dis (2015) 38(3):321–4. 10.1111/jfd.12248 24649885

[52] Ramirez R Gomez FA Marshall SH The infection process of Piscirickettsia salmonis in fish macrophages is dependent upon interaction with host-cell clathrin and actin. FEMS Microbiol Lett (2015) 362(1):1–8. 10.1093/femsle/fnu012 25790493

[53] Oliver C Sánchez P Valenzuela K Hernández M Pontigo JP Rauch MC Subcellular Location of Piscirickettsia salmonis Heat Shock Protein 60 (Hsp60) Chaperone by Using Immunogold Labeling and Proteomic Analysis. Microorganisms (2020) 8(1):117. 10.3390/microorganisms8010117 PMC7023422 31952216

[54] Hernandez AJ Romero A Gonzalez-Stegmaier R Dantagnan P The effects of supplemented diets with a phytopharmaceutical preparation from herbal and macroalgal origin on disease resistance in rainbow trout against Piscirickettsia salmonis. Aquaculture (2015) 454(1):109–17. 10.1016/j.aquaculture.2015.12.016

[55] Mickuviene I Kirveliene V Juodka B Experimental survey of non-clonogenic viability assays for adherent cells in vitro. Toxicol Vitro (2004) 18(5):639–48. 10.1016/j.tiv.2004.02.001 15251182

[56] Butler RE Brodin P Jang J Jang MS Robertson BD Gicquel B The balance of apoptotic and necrotic cell death in Mycobacterium tuberculosis infected macrophages is not dependent on bacterial virulence. PloS One (2012) 7(10):e47573. 10.1371/journal.pone.0047573 23118880 PMC3484146

[57] Yanez AJ Silva H Valenzuela K Pontigo JP Godoy M Troncoso J Two novel blood-free solid media for the culture of the salmonid pathogen Piscirickettsia salmonis. J Fish Dis (2013) 36(6):587–91. 10.1111/jfd.12034 23173561

[58] Kumar Y Valdivia RH Leading a sheltered life: intracellular pathogens and maintenance of vacuolar compartments. Cell Host Microbe (2009) 5(6):593–601. 10.1016/j.chom.2009.05.014 19527886 PMC2716004

[59] Elkin SR Lakoduk AM Schmid SL Endocytic pathways and endosomal trafficking: a primer. Wien Med Wochenschr (2016) 166(7-8):196–204. 10.1007/s10354-016-0432-7 26861668 PMC4873410

[60] Wang R Zhang Y Liu S Li C Sun L Bao L Analysis of 52 Rab GTPases from channel catfish and their involvement in immune responses after bacterial infections. Dev Comp Immunol (2014) 45(1):21–34. 10.1016/j.dci.2014.01.026 24513270

[61] Nepal A Wolfson DL Ahluwalia BS Jensen I Jørgensen J Iliev DB Intracellular distribution and transcriptional regulation of Atlantic salmon (Salmo salar) Rab5c, 7a and 27a homologs by immune stimuli. Fish Shellfish Immunol (2020) 99:119–29. 10.1016/j.fsi.2020.01.058 32014587

