# Supplementary figures and images for: Non-Specific Antibodies Induce Lysosomal Activation in Atlantic Salmon Macrophages Infected by Piscirickettsia salmonis
Source: Front Immunol. 2020 Nov 12;11:544718. doi: 10.3389/fimmu.2020.544718 (PMC7688784; doi:10.3389/fimmu.2020.544718)

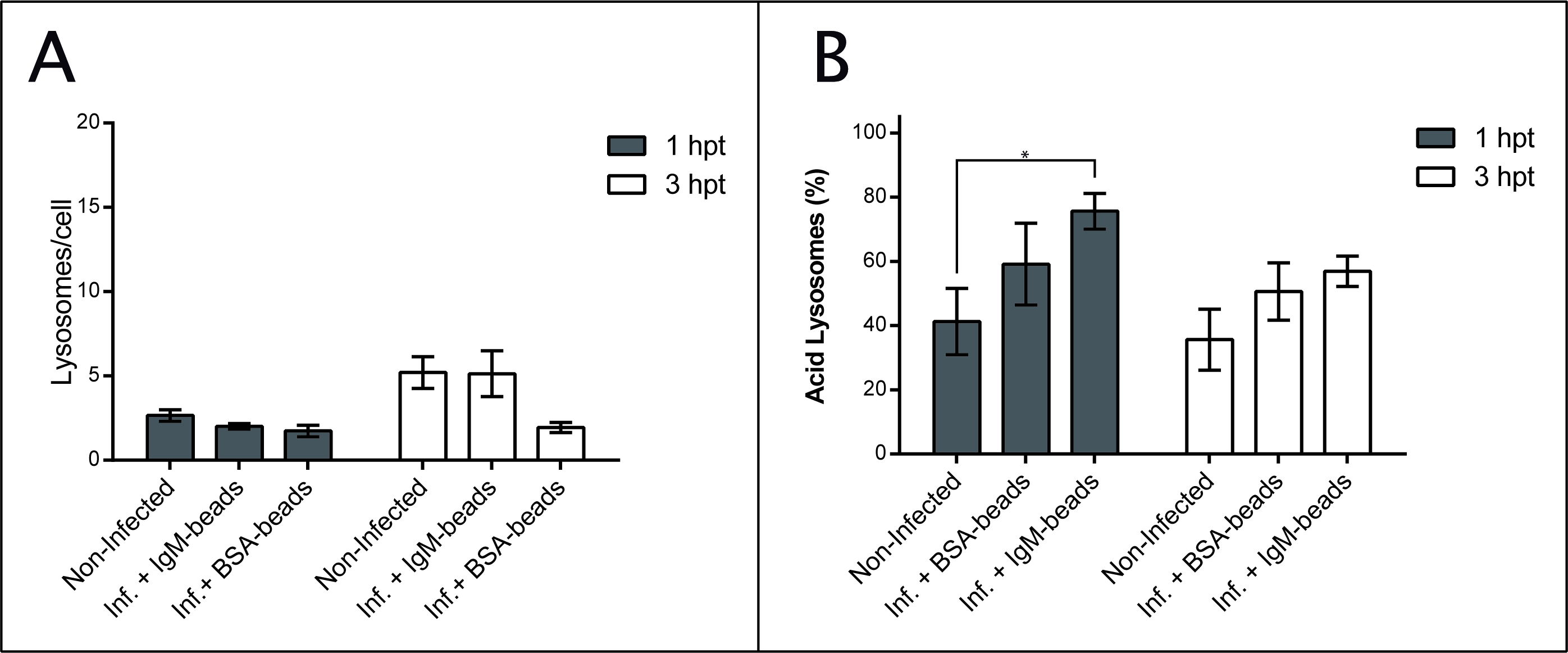

Supplement: Supplementary Figure 1 — Lysosomal quantification in infected macrophage-enriched cell cultures treated with IgM-beads and BSA-beads. Macrophage-enriched cell cultures were infected with P. salmonis at MOI of 10 bacteria/cell and analyzed at 1 and 3 hpt. The lysosomes were stained with the LSYB probe and quantified as acidic lysosomes or neutral-basic (NB) lysosomes. The data were normalized to the number of cells analyzed. (A) Total number of lysosomes per cell. (B) Percentage of acidic lysosomes for each condition. The statistical analysis was performed through parametric ANOVA with a Tukey multiple comparison test. Significant differences: *p < 0.05. [file Image_1.tif]

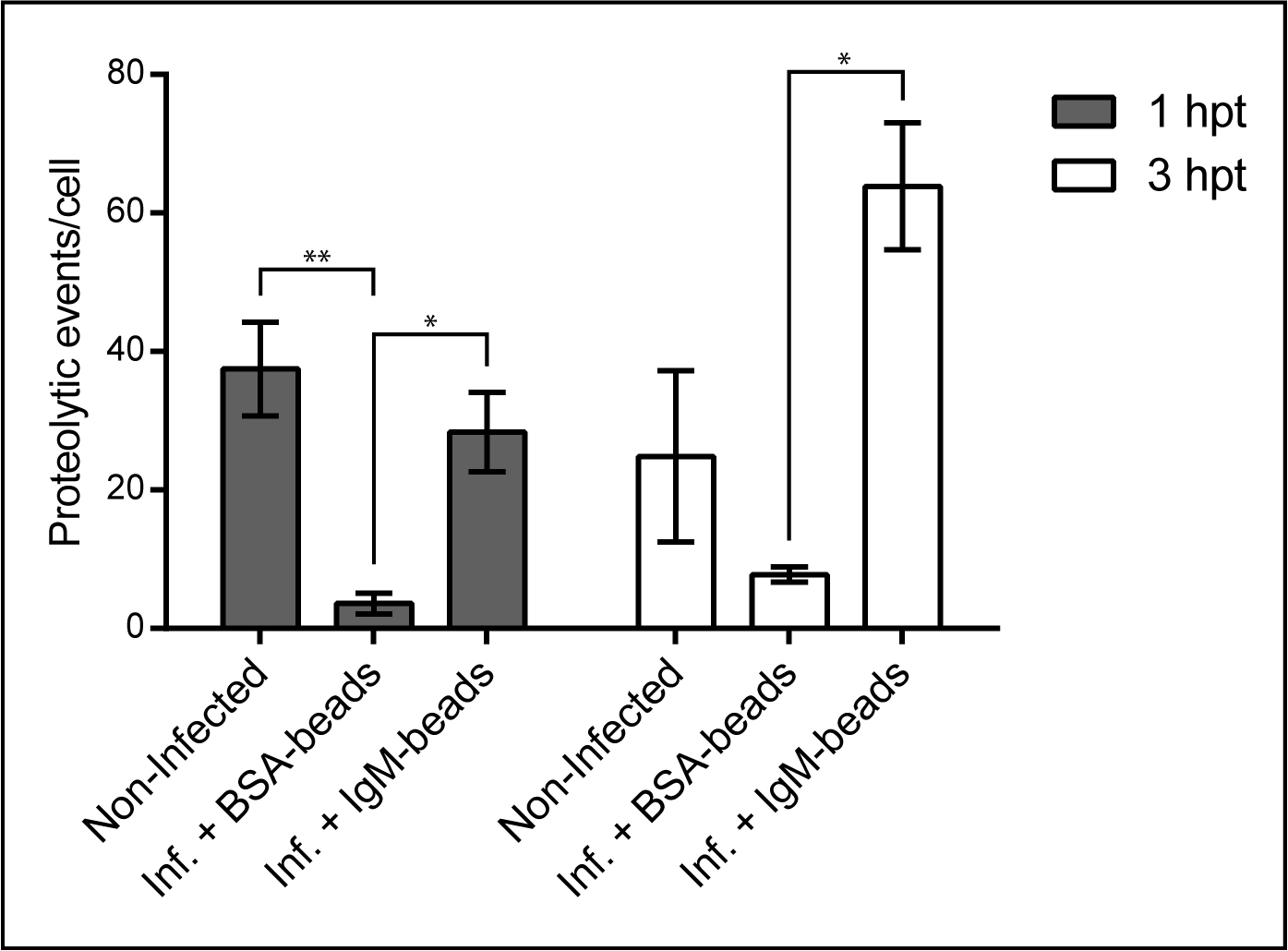

Supplement: Supplementary Figure 2 — Quantification of proteolytic events in infected macrophage-enriched cell cultures treated with IgM-beads and BSA-beads. Macrophage-enriched cell cultures were infected with P. salmonis at MOI of 10 bacteria/cell and analyzed at 1 and 3 hpt. The proteolytic events were detected using the DQ-BSATM Green probe and data were quantified and normalized to the number of cells analyzed for each condition. The statistical analysis was performed through a parametric ANOVA with a Tukey multiple comparison test. Significant differences: *p <0.05, **p < 0.01. [file Image_2.tif]

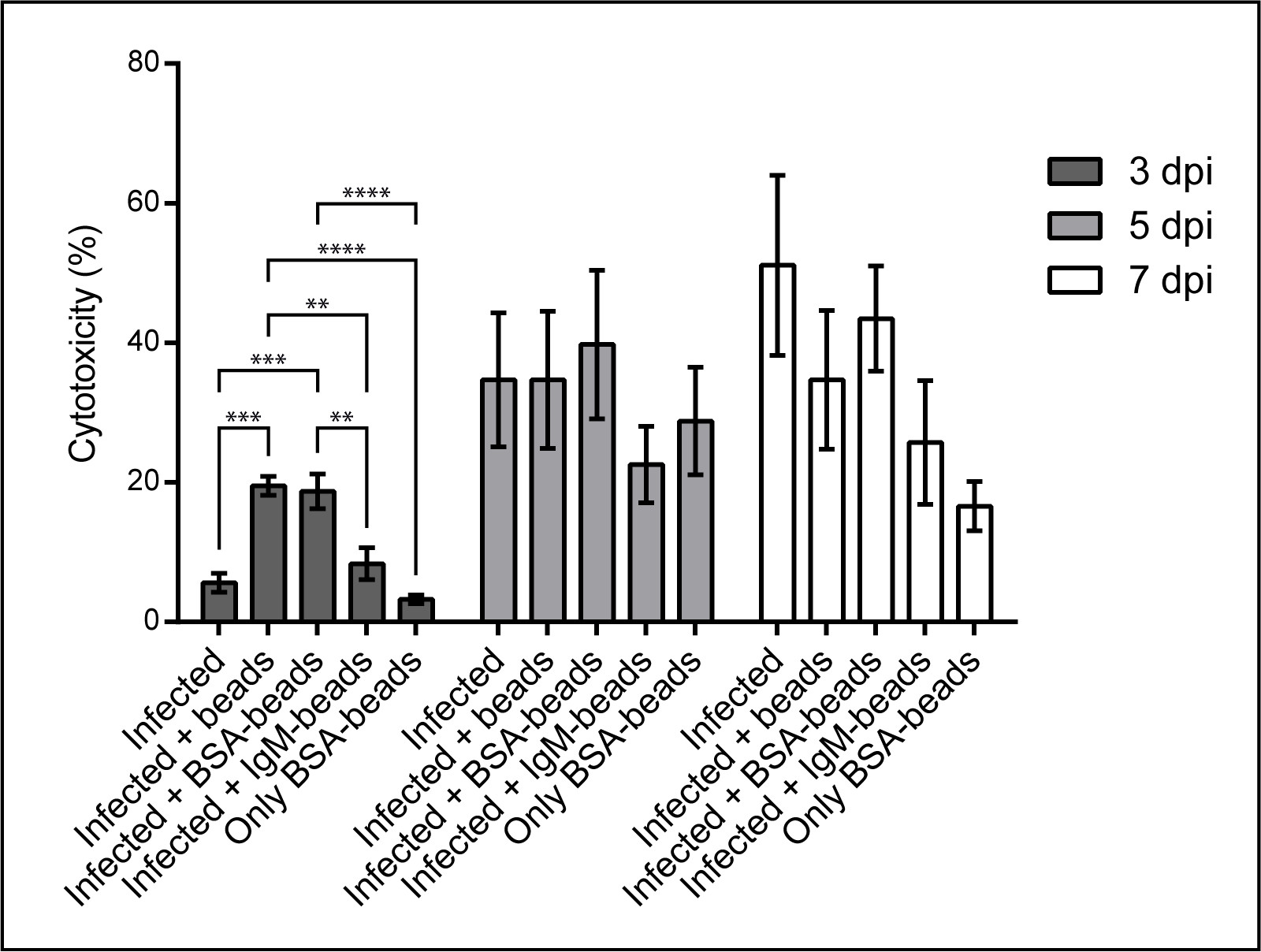

Supplement: Supplementary Figure 3 — Evaluation of cytotoxicity in infected macrophage-enriched cell cultures treated with IgM-beads and BSA-beads. Macrophage-enriched cell cultures obtained from S. salar were infected with P. salmonis at MOI of 10 bacteria/cell and treated with IgM-beads. The cytotoxicity was evaluated at 3, 5 and 7 dpi by the detection of LDH release into the extracellular medium. The statistical analysis was performed through a parametric ANOVA with a Tukey multiple comparison test. Significant differences: *p <0.05, **p < 0.01, ***p<0.001, **** p<0.0001. [file Image_3.tif]
