# Supplementary material for: Non-Specific Antibodies Induce Lysosomal Activation in Atlantic Salmon Macrophages Infected by Piscirickettsia salmonis
Source: Front Immunol. 2020 Nov 12;11:544718. doi: 10.3389/fimmu.2020.544718 (PMC7688784; doi:10.3389/fimmu.2020.544718)
Supplement: Supplementary file 4 [file DataSheet_1.docx]

Supplementary Data

Supplementary Table 1: Composition and concentrations of supplements in culture medium.

| Supplement | Components | Supplier | Final concentration |
| --- | --- | --- | --- |
| 1 | 2-mercaptoethanol | Gibco, Thermo Scientific | 36 µM |
|  | Fetal bovine serum | HyClone, GE Healthcare Life Sciences | 3% (v/v) |
|  | Penicillin/streptomycin | Corning | 200 U/mL/200 µg/mL |
|  | amphotericin B | Corning | 2.5 mg/mL |
|  | sodic heparin | Leo Pharma | 24.7 UI/mL |
| 2 | 2-mercaptoethanol | Gibco, Thermo Scientific, | 36 µM |
|  | Fetal bovine serum | HyClone, GE Healthcare Life Sciences | 10% (v/v) |
|  | Penicillin/streptomycin | Corning | 200 U/mL/200 µg/mL |
|  | amphotericin B | Corning | 2.5 mg/mL |
| 3 | 2-mercaptoethanol | Gibco, Thermo Scientific | 36 µM |
|  | Fetal bovine serum | HyClone, GE Healthcare Life Sciences | 10% (v/v) |
| 4 | Fetal bovine serum | HyClone, GE Healthcare Life Sciences | 10% (v/v) |
|  | L-glutamine | Gibco, Thermo Scientific | 4 mM |
|  | 2-mercaptoethanol | Gibco, Thermo Scientific | 5 nM |
|  | Gentamicin | US Biological | 50 mg/mL |
| 5 | Fetal bovine serum | HyClone, GE Healthcare Life Sciences | 10% (v/v) |
|  | HEPES buffer | Corning | 10 mM |
|  | Non-essential amino acids | Corning | 1% (v/v) |

Supplementary Table 2: Number of lysosomes/cell observed in macrophage-enriched cell cultures. Values expressed in Mean ± SEM.

|  | 1 hpt | 3 hpt |
| --- | --- | --- |
| Non-Infected | 2.6 ± 0.3 | 5.2 ± 0.9 |
| Infected | 6.1 ± 1.3 | 6.0 ± 1.0 |
| P. salmonis[In] | 5.5 ± 0.9 | 15.1 ± 2.7 |
| Inf. + IgM-beads | 2.0 ± 0.1 | 5.1 ± 1.3 |
| Inf. + BSA-beads | 1.7 ± 0.3 | 1.9 ± 0.3 |

Supplementary Table 3: Percentage of acidic lysosomes in macrophage-enriched cell cultures. Values expressed in Mean ± SEM.

|  | 1 hpt | 3 hpt |
| --- | --- | --- |
| Non-Infected | 41.2 ± 10.3 | 35.6 ± 9.5 |
| Inf*e*cted | 73.2 ± 3.3 | 38.3 ± 5.8 |
| P. salmonis[In] | 76.7 ± 7.5 | 75.9 ± 8.5 |
| Inf. + IgM-beads | 75.6 ± 5.6 | 56.9 ± 4.7 |
| Inf. + BSA-beads | 59.1 ± 12.7 | 50.6 ± 8.9 |

Supplementary Table 4: Quantification of proteolytic events in macrophage-enriched cell cultures. Values expressed in Mean ± SEM.

|  | 1 hpt | 3 hpt |
| --- | --- | --- |
| Non-Infected | 37.5 ± 6.8 | 24.9 ± 12.4 |
| Infected | 16.1 ± 1.8 | 3.0 ± 1.7 |
| P. salmonis[In] | 30.2 ± 10.3 | 72.5 ± 16.2 |
| Inf. + IgM-beads | 28.4 ± 5.7 | 63.8 ± 9.2 |
| Inf. + BSA-beads | 3.6 ± 1.5 | 7.8 ± 1.1 |

Supplementary Table 5: Quantification of cytotoxicity in SHK-1 cells measured by LDH-release. Values expressed in Mean ± SEM.

|  | 3 dpi | 5 dpi | 7 dpi |
| --- | --- | --- | --- |
| Infected | 5.6 ± 1.3 | 34.6 ± 9.6 | 51.1 ± 12.9 |
| Infected + beads | 19.5 ± 1.4 | 34.7 ± 9.8 | 34.7 ± 9.9 |
| Infected + BSA-beads | 18.7 ± 2.5 | 39.8 ± 10.7 | 43.5 ± 7.5 |
| Infected + IgM-beads | 8.4 ± 2.3 | 22.6 ± 5.5 | 25.6 ± 8.9 |
| Only IgM-beads | 8.3 ± 2.4 | 23.7 ± 7.2 | 24.5 ± 4.5 |
| Only BSA-beads | 3.3 ± 0.6 | 28.6 ± 7.7 | 16.6 ± 3.5 |

Supplementary Table 6: Quantification of *P. salmonis* infecting macrophage-enriched cell cultures. Values expressed in Mean ± SEM.

| **16S rDNA 72 hpi** | | |
| --- | --- | --- |
|  | Intracellular | Extracellular |
| Infected | 3.5 x 10^3^ ± 0.2 x 10^3^ | 2.8 x 10^3^ ± 0.2 x 10^3^ |
| Inf. + BSA-beads | 3.8 x 10^3^ ± 0.1 x 10^3^ | 3.1 x 10^3^ ± 0.2 x 10^3^ |
| Inf. + IgM-beads | 1.8 x 10^3^ ± 0.1 x 10^3^ | 1.5 x 10^3^ ± 0.1 x 10^3^ |
| **16S rDNA 120 hpi** | | |
|  | Intracellular | Extracellular |
| Infected | 1.9 x 10^4^ ± 1.0 x 10^3^ | 3.7 x 10^4^ ± 0.8 x 10^3^ |
| Inf. + BSA-beads | 2.0 x 10^4^ ± 1.2 x 10^3^ | 3.5 x 10^4^ ± 1.3 x 10^3^ |
| Inf. + IgM-beads | 1.2 x 10^4^ ± 0.7 x 10^3^ | 2.2 x 10^4^ ± 1.2 x 10^3^ |
|  | | |
| **CFU 72 hpi** | | |
|  | Intracellular | Extracellular |
| Infected | 0.074 ± 0.003 | 0.075 ± 0.005 |
| Inf. + BSA-beads | 0.085 ± 0.005 | 0.066 ± 0.006 |
| Inf. + IgM-beads | 0.042 ± 0.003 | 0.031 ± 0.002 |
| **CFU 120 hpi** | | |
|  | Intracellular | Extracellular |
| Infected | 0.503 ± 0.053 | 0.766 ± 0.043 |
| Inf. + BSA-beads | 0.466 ± 0.044 | 0.783 ± 0.065 |
| Inf. + IgM-beads | 0.325 ± 0.024 | 0.488 ± 0.033 |
